# Stress in Academic and Athletic Performance in Collegiate Athletes: A Narrative Review of Sources and Monitoring Strategies

**DOI:** 10.3389/fspor.2020.00042

**Published:** 2020-05-08

**Authors:** Marcel Lopes Dos Santos, Melissa Uftring, Cody A. Stahl, Robert G. Lockie, Brent Alvar, J. Bryan Mann, J. Jay Dawes

**Affiliations:** ^1^School of Kinesiology, Applied Health and Recreation, Oklahoma State University, Stillwater, OK, United States; ^2^Department of Kinesiology, California State University, Fullerton, CA, United States; ^3^Department of Kinesiology, Point Loma Nazarene University, San Diego, CA, United States; ^4^Department of Kinesiology and Sport Sciences, University of Miami, Miami, FL, United States

**Keywords:** stress, load management, academic stress, stress management, injury

## Abstract

College students are required to manage a variety of stressors related to academic, social, and financial commitments. In addition to the burdens facing most college students, collegiate athletes must devote a substantial amount of time to improving their sporting abilities. The strength and conditioning professional sees the athlete on nearly a daily basis and is able to recognize the changes in performance and behavior an athlete may exhibit as a result of these stressors. As such, the strength and conditioning professional may serve an integral role in the monitoring of these stressors and may be able to alter training programs to improve both performance and wellness. The purpose of this paper is to discuss stressors experienced by collegiate athletes, developing an early detection system through monitoring techniques that identify the detrimental effects of stress, and discuss appropriate stress management strategies for this population.

## Introduction

The college years are a period of time when young adults experience a significant amount of change and a variety of novel challenges. Academic performance, social demands, adjusting to life away from home, and financial challenges are just a few of the burdens college students must confront (Humphrey et al., 2000; Paule and Gilson, 2010; Aquilina, 2013). In addition to these stressors, collegiate athletes are required to spend a substantial amount of time participating in activities related to their sport, such as attending practices and training sessions, team meetings, travel, and competitions (Humphrey et al., 2000; López de Subijana et al., 2015; Davis et al., 2019; Hyatt and Kavazis, 2019). These commitments, in addition to the normal stress associated with college life, may increase a collegiate-athlete's risk of experiencing both physical and mental issues (Li et al., 2017; Moreland et al., 2018) that may affect their overall health and wellness. For these reasons, it is essential that coaches understand the types of stressors collegiate athletes face in order to help them manage the potentially deleterious effects stress may have on athletic and academic performance.

Strength and conditioning coaches are allied health care professionals whose primary job is to enhance fitness of individuals for the purpose of improving athletic performance (Massey et al., 2002, 2004, 2009). As such, many universities and colleges hire strength and conditioning coaches as part of their athletic staff to help athletes maximize their physical potential (Massey et al., 2002, 2004, 2009). Strength and conditioning coaches strive to increase athletic performance by the systematic application of physical stress to the body via resistance training, and other forms of exercise, to yield a positive adaptation response (Massey et al., 2002, 2004, 2009). For this reason, they need to understand and to learn how to manage athletes' stress. Additionally, based on the cumulative nature of stress, it is important that both mental and emotional stressors are also considered in programming. It is imperative that strength and conditioning coaches are aware of the multitude of stressors collegiate athletes encounter, in order to incorporate illness and injury risk management education into their training programs (Radcliffe et al., 2015; Ivarsson et al., 2017).

Based on the large number of contact hours strength and conditioning coaches spend with their athletes, they are in an optimal position to assist athletes with developing effective coping strategies to manage stress. By doing so, strength and conditioning coaches may be able to help reach the overarching goal of improving the health, wellness, fitness, and performance of the athletes they coach. The purpose of this review article is to provide the strength and conditioning professional with a foundational understanding of the types of stressors collegiate athletes may experience, and how these stressors may impact mental health and athletic performance. Suggestions for assisting athletes with developing effective coping strategies to reduce potential physiological and psychological impacts of stress will also be provided.

### Stress and the Stress Response

In its most simplistic definition, stress can be described as a state of physical and psychological activation in response to external demands that exceed one's ability to cope and requires a person to adapt or change behavior. As such, both cognitive or environmental events that trigger stress are called stressors (Statler and DuBois, 2016). Stressors can be acute or chronic based on the duration of activation. Acute stressors may be defined as a stressful situation that occurs suddenly and results in physiological arousal (e.g., increase in hormonal levels, blood flow, cardiac output, blood sugar levels, pupil and airway dilation, etc.) (Selye, 1976). Once the situation is normalized, a cascade of hormonal reactions occurs to help the body return to a resting state (i.e., homeostasis). However, when acute stressors become chronic in nature, they may increase an individual's risk of developing anxiety, depression, or metabolic disorders (Selye, 1976). Moreover, the literature has shown that cumulative stress is correlated with an increased susceptibility to illness and injury (Szivak and Kraemer, 2015; Mann et al., 2016; Hamlin et al., 2019). The impact of stress is individualistic and subjective by nature (Williams and Andersen, 1998; Ivarsson et al., 2017). Additionally, the manner in which athletes respond to a situational or environmental stressor is often determined by their individual perception of the event (Gould and Udry, 1994; Williams and Andersen, 1998; Ivarsson et al., 2017). In this regard, the athlete's perception can either be positive (eustress) or negative (distress). Even though they both cause physiological arousal, eustress also generates positive mental energy whereas distress generates anxiety (Statler and DuBois, 2016). Therefore, it is essential that an athlete has the tools and ability to cope with these stressors in order to have the capacity to manage both acute and chronic stress. As such, it is important to understand the types of stressors collegiate athletes are confronted with and how these stressors impact an athlete's performance, both athletically and academically.

## Methods

### Literature Search/Data Collection

The articles included in this review were identified via online databases PubMed, MEDLINE, and ISI Web of Knowledge from October 15th 2019 through January 15th 2020. The search strategy combined the keywords “academic stress,” “athletic stress,” “stress,” “stressor,” “college athletes,” “student athletes,” “collegiate athletes,” “injury,” “training,” “monitoring.” Duplicated articles were then removed. After reading the titles and abstracts, all articles that met the inclusion criteria were considered eligible for inclusion in the review. Subsequently, all eligible articles were read in their entirety and were either included or removed from the present review.

### Inclusion Criteria

The studies included met all the following criteria: (i) published in English-language journals; (ii) targeted college athletes; (iii) publication was either an original research paper or a literature review; (iv) allowed the extraction of data for analysis.

### Data Analysis

Relevant data regarding participant characteristics (i.e., gender, academic status, sports) and study characteristics were extracted. Articles were analyzed and divided into two separate sections based on their specific topics: Academic Stress and Athletic Stress. Then, strategies for monitoring and workload management are discussed in the final section.

## Academic Stress

Fundamentally, collegiate athletes have two major roles they must balance as part of their commitment to a university: being a college student and an athlete. Academic performance is a significant source of stress for most college students (Aquilina, 2013; López de Subijana et al., 2015; de Brandt et al., 2018; Davis et al., 2019). This stress may be further compounded among collegiate athletes based on their need to be successful in the classroom, while simultaneously excelling in their respective sport (Aquilina, 2013; López de Subijana et al., 2015; Huml et al., 2016; Hamlin et al., 2019). Davis et al. (2019) conducted surveys on 173 elite junior alpine skiers and reported significant moderate to strong correlations between perceived stress and several variables including depressed mood (*r* = 0.591), sleep disturbance (*r* = 0.459), fatigue (*r* = 0.457), performance demands (*r* = 0.523), and goals and development (*r* = 0.544). Academic requirements were the highest scoring source of stress of all variables and was most strongly correlated with perceived stress (*r* = 0.467). Interestingly, it was not academic rigor that was viewed by the athletes as the largest source of direct stress; rather, the athletes surveyed reported time management as being their biggest challenge related to academic performance (Davis et al., 2019). This further corroborates the findings of Hamlin et al. (2019). The investigators reported that during periods of the academic year in which levels of perceived academic stress were at their highest, students had trouble managing sport practices and studying. These stressors were also associated with a decrease in energy levels and overall sleep quality. These factors may significantly increase the collegiate athlete's susceptibility to illness and injury (Hamlin et al., 2019). For this reason, coaches should be aware of and sensitive to the stressors athletes experience as part of the cyclical nature of the academic year and attempt to help athletes find solutions to balancing athletic and academic demands.

According to Aquilina (2013), collegiate athletes tend to be more committed to sports development and may view their academic career as a contingency plan to their athletic career, rather than a source of personal development. As a result, collegiate athletes often, but certainly not always, prioritize athletic participation over their academic responsibilities (Miller and Kerr, 2002; Cosh and Tully, 2014, 2015). Nonetheless, scholarships are usually predicated on both athletic and academic performance. For instance, the National Collegiate Athletic Association (NCAA) requires collegiate athletes to achieve and maintain a certain grade point average (GPA). Furthermore, they are also often required to also uphold a certain GPA to maintain an athletic scholarship. The pressure to maintain both high levels of academic and athletic performance may increase the likelihood of triggering mental health issues (i.e., anxiety and depression) (Li et al., 2017; Moreland et al., 2018).

Mental health issues are a significant concern among college students. There has been an increased emphasis placed on the mental health of collegiate athletes in recent years (Petrie et al., 2014; Li et al., 2017, 2019; Reardon et al., 2019). Based on the 2019 National College Health Assessment survey from the American College Health Association (ACHA) consisting of 67,972 participants, 27.8% of college students reported anxiety, and 20.2% reported experiencing depression which negatively affected their academic performance (American College Health Association American College Health Association-National College Health Assessment II, 2019). Approximately 65.7% (50.7% males and 71.8% females) reported feeling overwhelming anxiety in the past 12 months, and 45.1% (37.1% males and 47.6% females) reported feeling so depressed that it was difficult for them to function. However, only 24.3% (13% males and 28.4% females) reported being diagnosed and treated by a professional in the past 12 months. Collegiate athletes are not immune to these types of issues. According to information presented by the NCAA, many certified athletic trainers anecdotally state that anxiety is an issue affecting the collegiate-athlete population (NCAA, 2014). However, despite the fact that collegiate athletes are exposed to numerous stressors, they are less likely to seek help at a university counseling center than non-athletes (NCAA, 2014), which could be related to stigmas that surround mental health services (NCAA, 2014; Kaier et al., 2015; Egan, 2019). This not only has significant implications related to their psychological well-being, but also their physiological health, and consequently their performance. For instance, in a study by Li et al. (2017) it was found that NCAA Division I athletes who reported preseason anxiety symptoms had a 2.3 times greater injury incidence rate compared to athletes who did not report. This same study discovered that male athletes who reported preseason anxiety and depression had a 2.1 times greater injury incidence, compared to male athletes who did not report symptoms of anxiety and depression. (Lavallée and Flint, 1996) also reported a correlation between anxiety and both injury frequency and severity among college football players (*r* = 0.43 and *r* = 0.44, respectively). In their study, athletes reporting high tension/anxiety had a higher rate of injury. It has been suggested that the occurrence of stress and anxiety may cause physiological responses, such as an increase in muscle tension, physical fatigue, and a decrease in neurocognitive and perception processes that can lead to physical injuries (Ivarsson et al., 2017). For this reason, it is reasonable to consider that academic stressors may potentiate effects of stress and result in injury and illness in collegiate athletes.

Periods of more intense academic stress increase the susceptibility to illness or injury (Mann et al., 2016; Hamlin et al., 2019; Li et al., 2019). For example, Hamlin et al. (2019) investigated levels of perceived stress, training loads, injury, and illness incidence in 182 collegiate athletes for the period of one academic year. The highest levels of stress and incidence of illness arise during the examination weeks occurring within the competitive season. In addition, the authors also reported the odds ratio, which is the occurrence of the outcome of interest (i.e., injury), based off the given exposure to the variables of interest (i.e., perceived mood, sleep duration, increased academic stress, and energy levels). Based on a logistic regression, they found that each of the four variables (i.e., mood, energy, sleep duration, and academic stress) was related to the collegiate athletes' likelihood to incur injuries. In summary, decreased levels of perceived mood (odds ratio of 0.89, 0.85–0.0.94 CI) and sleep duration (odds ratio of 0.94, 0.91–0.97 CI), and increased academic stress (odds ratio of 0.91, 0.88–0.94 CI) and energy levels (odds ratio of 1.07, 1.01–1.14 CI), were able to predict injury in these athletes. This corroborates Mann et al. (2016) who found NCAA Division I football athletes at a Bowl Championship Subdivision university were more likely to become ill or injured during an academically stressful period (i.e., midterm exams or other common test weeks) than during a non-testing week (odds ratio of 1.78 for high academic stress). The athletes were also less likely to get injured during training camp (odds ratio of 3.65 for training camp). Freshmen collegiate athletes may be especially more susceptible to mental health issues than older students. Their transition includes not only the academic environment with its requirements and expectations, but also the adaptation to working with a new coach and teammates. In this regard, Yang et al. (2007) found an increase in the likelihood of depression that freshmen athletes experienced, as these freshmen were 3.27 times more likely to experience depression than their older teammates. While some stressors are recurrent and inherent in academic life (e.g., attending classes, homework, etc.), others are more situational (e.g., exams, midterms, projects) and may be anticipated by the strength and conditioning coach.

## Athletic Stress

The domain of athletics can expose collegiate athletes to additional stressors that are specific to their cohort (e.g., sport-specific, team vs. individual sport) (Aquilina, 2013). Time spent training (e.g., physical conditioning and sports practice), competition schedules (e.g., travel time, missing class), dealing with injuries (e.g., physical therapy/rehabilitation, etc.), sport-specific social support (e.g., teammates, coaches) and playing status (e.g., starting, non-starter, being benched, etc.) are just a few of the additional challenges collegiate athletes must confront relative to their dual role of being a student and an athlete (Maloney and McCormick, 1993; Scott et al., 2008; Etzel, 2009; Fogaca, 2019). Collegiate athletes who view the demands of stressors from academics and sports as a positive challenge (i.e., an individual's self-confidence or belief in oneself to accomplish the task outweighs any anxiety or emotional worry that is felt) may potentially increase learning capacity and competency (NCAA, 2014). However, when these demands are perceived as exceeding the athlete's capacity, this stress can be detrimental to the student's mental and physical health as well as to sport performance (Ivarsson et al., 2017; Li et al., 2017).

As previously stated, time management has been shown to be a challenge to collegiate athletes. The NCAA rules state that collegiate athletes may only engage in required athletic activities for 4 h per day and 20 h/week during in-season and 8 h/week during off-season throughout the academic year. Although these rules have been clearly outlined, the most recent NCAA GOALS (2016) study reported alarming numbers regarding time commitment to athletic-related activities. Data from over 21,000 collegiate athletes from 600 schools across Divisions I, II, and III were included in this study. Although a breakdown of time commitments was not provided, collegiate athletes reported dedicating up to 34 h per week to athletics (e.g., practices, weight training, meetings with coaches, tactical training, competitions, etc.), in addition to spending between 38.5 and 40 h per week working on academic-related tasks. This report also showed a notable trend related to athletes spending an increase of ~2 more athletics-related hours per week compared to the 2010 GOALS study, along with a decrease of 2 h of personal time (from 19.5 h per week in 2010 to 17.1 in 2015). Furthermore, ~66% of Division I and II and 50% of Division III athletes reported spending as much or more time in their practices during the off-season as during the competitive season (DTHOMAS, 2013). These numbers show how important it is for collegiate athletes to develop time management skills to be successful in both academics and athletics. Overall, most collegiate athletes have expressed a need to find time to enjoy their college experience outside of athletic obligations (Paule and Gilson, 2010). Despite that, because of the increasing demand for excellence in academics and athletics, collegiate athletes' free time with family and friends is often scarce (Paule and Gilson, 2010). Consequently, trainers, coaches, and teammates will likely be the primary source of their weekly social interactivity.

Social interactions within their sport have also been found to relate to factors that may impact an athlete's perceived stress. Interactions with coaches and trainers can be effective or deleterious to an athlete. Effective coaching includes a coaching style that allows for a boost of the athlete's motivation, self-esteem, and efficacy in addition to mitigating the effects of anxiety. On the other hand, poor coaching (i.e., the opposite of effective coaching) can have detrimental psychological effects on an athlete (Gearity and Murray, 2011). In a closer examination of the concept of poor coaching practices, Gearity and Murray (2011) interviewed athletes about their experiences of receiving poor coaching. Following analysis of the interviews, the authors identified the main themes of the “coach being uncaring and unfair,” “practicing poor teaching inhibiting athlete's mental skills,” and “athlete coping.” They stated that inhibition of an athlete's mental skills and coping are associated with the psychological well-being of an athlete. Also, poor coaching may result in mental skills inhibition, distraction, insecurity, and ultimately team division (Gearity and Murray, 2011). This combination of factors may compound the negative impacts of stress in athletes and might be especially important for in injured athletes.

Injured athletes have previously been reported to have elevated stress as a result of heightened worry about returning to pre-competition status (Crossman, 1997), isolation from teammates if the injury is over a long period of time (Podlog and Eklund, 2007) and/or reduced mood or depressive symptoms (Daly et al., 1995). In addition, athletes who experience prolonged negative thoughts may be more likely to have decreased rehabilitation attendance or adherence, worse functional outcomes from rehabilitation (e.g., on measures of proprioception, muscular endurance, and agility), and worse post-injury performance (Brewer, 2012).

## Monitoring Considerations

In addition to poor coaching, insufficient workload management can hinder an athlete's ability to recover and adapt to training, leading to fatigue accumulation (Gabbett et al., 2017). Excessive fatigue can impair decision-making ability, coordination and neuromuscular control, and ultimately result in overtraining and injury (Soligard et al., 2016). For instance, central fatigue was found to be a direct contributor to anterior cruciate ligament injuries in soccer players (Mclean and Samorezov, 2009). Introducing monitoring tools may serve as a means to reduce the detrimental effects of stress in collegiate athletes. Recent research on relationships between athlete workloads, injury, and performance has highlighted the benefits of athlete monitoring (Drew and Finch, 2016; Jaspers et al., 2017).

Athlete monitoring is often assessed with the measuring and management of workload associated with a combination of sport-related and non-sport-related stressors (Soligard et al., 2016). An effective workload management program should aim to detect excessive fatigue, identify its causes, and constantly adapt rest, recovery, training, and competition loads respectively (Soligard et al., 2016). The workload for each athlete is based off their current levels of physical and psychological fatigue, wellness, fitness, health, and recovery (Soligard et al., 2016). Accumulation of situational or physical stressors will likely result in day-to-day fluctuations in the ability to move external loads and strength train effectively (Fry and Kraemer, 1997). Periods of increased academic stress may cause increased levels of fatigue, which can be identified by using these monitoring tools, thereby assisting the coaches with modulating the workload during these specific periods. Coaches who plan to incorporate monitoring and management strategies must have a clear understanding of what they want to achieve from athlete monitoring (Gabbett et al., 2017; Thornton et al., 2019).

### Monitoring External Loads

External load refers to the physical work (e.g., number of sprints, weight lifted, distance traveled, etc.) completed by the athlete during competition, training, and activities of daily living (Soligard et al., 2016). This type of load is independent of the athlete's individual characteristics (Wallace et al., 2009). Monitoring external loading can aid in the designing of training programs which mimic the external load demands of an athlete's sport, guide rehabilitation programs, and aid in the detection of spikes in external load that may increase the risk of injury (Clubb and McGuigan, 2018).

The means of quantifying external load can involve metrics as simple as pitch counts in baseball and softball (Fleisig and Andrews, 2012; Shanley et al., 2012) or quantifying lifting session training loads (e.g., sum value of weight lifted during an exercise x number of repetitions × the number of sets). Neuromuscular function testing is another more common way of analyzing external load. This is typically done using such measures such as the counter movement jump, squat jump, or drop jump. A force platform can be used to measure a myriad of outcomes (e.g., peak power, ground contact time, time to take-off, reactive strength index, and jump height), or simply measure jump height in a more traditional manner. Jumping protocols, such as the countermovement jump, have been adopted to examine the recovery of neuromuscular function after athletic competition with significant decreases for up to 72 h commonly reported (Andersson et al., 2008; Magalhães et al., 2010; Twist and Highton, 2013). (Gathercole et al., 2015) found reductions in 18 different neuromuscular variables in collegiate athletes following a fatiguing protocol. The variables of eccentric duration, concentric duration, total duration, time to peak force/power, and flight time:contraction time ratio, derived from a countermovement jump were deemed suitable for detecting neuromuscular fatigue with the rise in the use of technology for monitoring, certain sports have adopted specific software that can aid in the monitoring of stress. For example, power output can be measured using devices such as SRM™ or PowerTap™ in cycling (Jobson et al., 2009). This data can be analyzed to provide information such as average power or normalized power. The power output can then be converted into a Training Stress Score™ via commercially available software (Marino, 2011). More sophisticated measures of external load may involve the use of wearable technology devices such as Global Positioning System (GPS) devices, accelerometers, magnetometer, and gyroscope inertial sensors (Akenhead and Nassis, 2016). These devices can quantify external load in several ways, such as duration of movement, total distance covered, speed of movement, acceleration, and decelerations, as well as sport specific movement such as number and height of jumps, number of tackles, or breakaways, etc. (Akenhead and Nassis, 2016). The expansion of marketing of wearable devices has been substantial; however, there are questions of validity and reliability related to external load tracking limitations related to proprietary metrics, as well as the overall cost that should be considered when considering the adoption of such devices (Aughey et al., 2016; Torres-Ronda and Schelling, 2017).

### Monitoring Internal Loads

While external load may provide information about an athlete's performance capacity and work completed, it does not provide clear evidence of how athletes are coping with and adapting to the external load (Halson, 2014). This type of information comes from the monitoring of internal loads. The term internal load refers to the individual physiological and psychological response to the external stress or load imposed (Wallace et al., 2009). Internal load is influenced by a number of factors such as daily life stressors, the environment around the athlete, and coping ability (Soligard et al., 2016). Indirect measures, such as the use of heart rate (HR) monitoring, and subjective measurements, such as perceived effort (i.e., ratings of perceived exertion), are examples of internal load monitoring. Using subjective measurement systems is a simple and practical method when dealing with large numbers of athletes (Saw et al., 2016; Nässi et al., 2017). Subjective reporting of training load (Rating of Perceived Exertion—RPE) (Coyne et al., 2018), Session Rating of Perceived Exertion—sRPE) (Coyne et al., 2018), perceived stress and recovery (Recovery Stress Questionnaire for Athletes—RESTQ-S), and psychological mood states (Profile of Mood States—POMS) have all been found to be a reliable indicator of training load (Robson-Ansley et al., 2009; Saw et al., 2016) and only take a few moments to complete. In addition, subjective measures can be more responsive to tracking changes or training responses in athletes than objective measures (Saw et al., 2016).

Heart rate (HR) monitoring is a common intrinsic measure of how the body is responding to stress. With training, the reduction of resting HR is typically a clear indication of the heart becoming more efficient and not having to beat as frequently. Alternately, increases of resting HR over time with a continuation of training may be an indicator of too much stress. Improper nutrition, such as regular or ongoing suboptimal intakes of vitamins or minerals, may result in increased ventilation and/or increased heart rate (Lukaski, 2004). It has been suggested that the additional stress may lead to parasympathetic hyperactivity, leading to an increase in resting HR (Statler and DuBois, 2016). This largely stems from research examining the sensitivity of various HR derived metrics, such as resting HR, HR variability (HRV), and HR recovery (HRR) to fluctuations in training load (Borresen and Ian Lambert, 2009). HRR in athlete monitoring is the rate of HR decline after the cessation of exercise. A common measure of HHR is the use of a 2 min step test followed by a 60 s HR measurement. The combination of the exercise (stress) on the cardiovascular system and then its subsequent return toward baseline has been used as an indicator of autonomic function and training status in athletes (Daanen et al., 2012). In collegiate athletes it was found that hydration status impacted HRR following moderate to hard straining sessions (Ayotte and Corcoran, 2018). Athletes who followed a prescription hydration plan performed better in the standing long jump, tracked objects faster, and showed faster HRR vs. athletes who followed their normal self-selected hydration plan (Ayotte and Corcoran, 2018). To date, HR monitoring and the various derivatives have mainly been successful in detecting changes in training load and performance in endurance athletes (Borresen and Ian Lambert, 2009; Lamberts et al., 2009; Thorpe et al., 2017). Although heart rate monitoring can provide additional physiological insight for aerobic sessions or events, it thus far has not been found to be an accurate measurement for quantifying internal load during many explosive, short duration anaerobic activities (Bosquet et al., 2008).

A multitude of studies have reported the reliability and validity of using RPE and sRPE across a range of training modalities (Foster, 1998; Impellizzeri et al., 2004; Sweet et al., 2004). This measure can be used to create a number of metrics such as session load (sRPE × duration in minutes), daily load (sum of all session loads for that day), weekly training load (sum of all daily training loads for entire week), monotony (standard deviation of weekly training load), and strain (daily or weekly training load × monotony) (Foster, 1998). Qualitative questionnaires that monitor stress and fatigue have been well-established as tools to use with athletes (see Table 1 for examples of commonly used questionnaires in research). Using short daily wellness questionnaires may allow coaches to generate a wellness score which then can be adjusted based off of the stress the athlete may be feeling to meet the daily load target (Foster, 1998; Robson-Ansley et al., 2009). However, strength and conditioning coaches need to be mindful that these questionnaires may require sports psychologist or other licensed professional to examine and provide the results. An alternative that may be better suited for strength and conditioning professionals to use could be to incorporate some of the themes of those questionnaires into programing.

**Table 1:** Overview of common tool/measures used by researchers to monitor training load.

**Category of training load**	**Variable examined**	**Tests or Methods of collection**	**References**
**External load**: defined as the work completed by the athlete, measured independently of their individual characteristics (Wallace et al., 2009)	Power output	Various devices	Pyne and Martin, 2011
	Neuromuscular function	Jump tests—CMJ or SJ performance	Twist and Highton, 2013
		Sprint performance	Twist and Highton, 2013
	Time-motion analysis	GPS tracking	Aughey, 2011; Halson, 2014
		Movement pattern analysis via digital video	Taylor et al., 2012; Halson (2014)
**Internal load:** related to physiological and psychological stress imposed (Wallace et al., 2009)	Perception of effort	Rating of Perceived Exertion (RPE)	Borresen and Ian Lambert, 2009
		Session Rating of Perceived Exertion (sRPE)	Foster, 1998
	Heart rate measures	Heart rate (HR)	Hopkins, 1991
		HR to RPE	Martin and Andersen, 2000
		HR recovery (HRR)	Daanen et al., 2012
		HR variability (HRV)	Plews et al., 2013
		Training Impulse (TRIMP)	Morton et al., 1990; Pyne and Martin, 2011
	Qualitative questionnaires^*^	Profile of Mood States (ROMS)	Morgan et al., 1987
		Recovery Stress Questionnaires for Athletes (REST-Q)	Kallus and Kellmann, 2016
		Daily Analysis of Life Demands for Athletes (DALDA)	Rushall, 1990
		Total Recovery Scale (TQR)	Kenttä and Hassmén, 1998

* *indicates variable/monitoring tool that is most appropriate for use by a sport psychologist or licensed psychologist*.

### A Multifaceted Approach

Dissociation between external and internal load units may be indicative of the state of fatigue of an athlete. Utilizing a monitoring system in which the athlete is able to make adjustments to their training loads in accordance with how they are feeling in that moment can be a useful tool for assisting the athlete in managing stress. Auto-regulation is a method of programming that allows for adjustments based on the results of one or more readiness tests. When implemented properly, auto regulation enables the coach or athlete to optimize training based on the athlete's given readiness for training on a particular day, thereby aiming to avoid potential overtraining (Kraemer and Fleck, 2018). Several studies have found that using movement velocity to designate resistance training intensities can result in significant improvements in maximal strength and athletic performance (Pareja-Blanco et al., 2014, 2017; Mann et al., 2015). Velocity based training allows the coach and athlete to view real time feedback for the given lifts, thereby allowing them to observe how the athlete is performing in that moment. If the athlete is failing to meet the prescribed velocity or the velocity drops greater than a predetermined amount between sets, then this should signal the coach to investigate. If there is a higher than normal amount of stress on that athlete for the day, that could be a potential reason. This type of combination style program of using a quantitative or objective measurement (s) and a subjective measure of wellness (qualitative questionnaire) has recently been reported to be an effective tool in monitoring individuals apart of a team (Starling et al., 2019). The subjective measure in this study was the readiness to train questionnaire (RTT-Q) and the objective measures were the HRR_6min_ test (specifically the HRR_60s_ = recorded as decrease in HR in the 60 s after termination of the test) to assess autonomic function and the standing long jump (SLJ) to measure neuromuscular function. The findings found that, based on the absolute typical error of measurement, the HRR_60s_ and SLJ could detect medium and large changes in fatigue and readiness. The test took roughly 8 min for the entire team, which included a group consisting of 24 college-age athletes. There are many other combinations of monitoring variables and strategies that coaches and athletes may utilize.

### Data Analysis – How to Utilize the Measures

Regardless of what type of monitoring tool a coach or athlete may incorporate, it is essential to understand how to analyze this data. There are excellent resources available which discuss this topic in great detail (Gabbett et al., 2017; Clubb and McGuigan, 2018; Thornton et al., 2019). This section will highlight two main conclusions from these sources and briefly describe two of the main statistical practices and concepts discussed. The use of z-scores or modified z-scores has been proposed as a method of detecting meaningful change in athlete data (Clubb and McGuigan, 2018; Thornton et al., 2019). For different monitoring tools listed in Table 1, the following formula would be an example of how to assess changes: (Athlete daily score—Baseline score)/Standard deviation of baseline. The baseline would likely be based off an appropriate period such as the scores across 2 weeks during the preseason.

In sports and sports science, the use of a magnitude-based inference (MBI) has been suggested as more appropriate and easier to understand when examining meaningful changes in athletic data, than null-hypothesis significance testing (NHST) (Buchheit, 2014). Additional methods to assess meaningful change that are similar to MBI are using standard deviation, typical error, effect sizes, smallest worthwhile change (SWC), and coefficient of variation (Thornton et al., 2019). It should be noted that all of these methods have faced criticism from sources such as statisticians. It is important to understand that the testing methods, measurements, and analysis should be based on the resources and intended goals from use, which will differ from every group and individual. Once identified, it is up to the practitioner to keep this system the same, in order to collect data that can then be examined to understand meaningful information for each setting (Thornton et al., 2019).

### Managing and Coping Strategies

Once the collegiate-athlete has been able to identify the need to balance their stress levels, the athlete may then need to seek out options for managing their stress. Coaches are be able to assist them by sharing information on health and wellness resources available for the students, both on and off campus. Another way a coach can potentially support their athletes is by establishing an open-door policy, wherein the team members feel comfortable approaching a member of the strength and conditioning staff in order to seek out resources for coping with challenges related to stress.

There are some basic skills that strength and conditioning coaches can teach (while staying within their scope of practice). Coaches can introduce their athletes to basic lifestyle concepts, such as practicing deep breathing techniques, positive self-talk, and developing healthy sleep habits (i.e., turning off their mobile devices 1 h before bed and aiming for 8 h of sleep each night, etc.). A survey of strength and conditioning practitioners by Radcliffe et al. (2015) found that strategies used by practitioners included a mix of cognitive and behavioral strategies, which was used as justification for recommending practitioners find opportunities to guide professional development toward awareness strategies. Practitioners reported using a wide variety of psychological skills and strategies, which following survey analysis, highlighted a significant emphasis on strategies that may influence athlete self-confidence and goal setting. Themes identified by Radcliffe et al. (2015) included confidence building, arousal management, and skill acquisition. Additionally, similar lower level themes that are connected (i.e., goal setting, increasing, or decreasing arousal intensities, self-talk, mental imagery) are all discussed in the 4th edition of the NSCA Essentials of Strength and Conditioning book (Haff et al., 2016). When the interventions aiming to improve mental health expand from basic concepts to mental training beyond a coach's scope, it would be pertinent for the coach to refer the collegiate-athlete to a sport psychology or other mental health consultant (Fogaca, 2019). Moreover, strength and conditioning coaches may find themselves in a position to become key players in facilitating management strategies for collegiate athletes, thereby guiding the athlete in their quest to learn how to best manage the mental and physical energy levels required in the quest for overall optimal performance (Statler and DuBois, 2016).

## Conclusion and Future Directions

This review article has summarized some of the ways that strength and conditioning professionals may be able to gain a better understanding of the types of stressors encountered by collegiate athletes, the impact these stressors may have on athletic performance, and suggestions for assisting athletes with developing effective coping strategies to reduce the potential negative physiological and psychological impacts of stress. It has been suggested that strategies learned in the context of training may have a carry-over effect into other areas such as competition. More education is needed in order for strength and conditioning professionals to gain a greater understanding of how to support their athletes with stress-management techniques and resources. Some ways to disseminate further education on stress-management tools for coaches to share with their athletes may include professional development events, such as conferences and clinics.

## Author Contributions

All of the authors have contributed to the development of the manuscript both in writing and conceptual development.

## Conflict of Interest

The authors declare that the research was conducted in the absence of any commercial or financial relationships that could be construed as a potential conflict of interest. The handling editor declared a past collaboration with one of the authors RL.
